# Identification of Nematicidal Constituents of *Notopterygium incisum* Rhizomes against *Bursaphelenchus xylophilus* and *Meloidogyne incognita*

**DOI:** 10.3390/molecules21101276

**Published:** 2016-09-23

**Authors:** Gai Liu, Daowan Lai, Qi Zhi Liu, Ligang Zhou, Zhi Long Liu

**Affiliations:** 1 Department of Entomology, China Agricultural University, Haidian District, Beijing 100193, China; gailiu@126.com (G.L.); lqzwyz@cau.edu.cn (Q.Z.L.); 2 Department of Plant Pathology, China Agricultural University, Haidian District, Beijing 100193, China; dwlai@cau.edu.cn (D.L.); lgzhou@cau.edu.cn (L.Z.)

**Keywords:** *Notopterygium incisum*, *Bursaphelenchus xylophilus*, *Meloidogyne incognita*, nematicidal activity

## Abstract

During a screening program for new agrochemicals from Chinese medicinal herbs, the ethanol extract of *Notopterygium incisum* rhizomes was found to possess strong nematicidal activity against the two species of nematodes, *Bursaphelenchus xylophilus* and *Meloidogyne incognita*. Based on bioactivity-guided fractionation, the four constituents were isolated from the ethanol extract and identified as columbianetin, falcarindiol, falcarinol, and isoimperatorin. Among the four isolated constituents, two acetylenic compounds, falcarindiol and falcarinol (2.20–12.60 μg/mL and 1.06–4.96 μg/mL, respectively) exhibited stronger nematicidal activity than two furanocoumarins, columbianetin, and isoimperatorin (21.83–103.44 μg/mL and 17.21–30.91 μg/mL, respectively) against the two species of nematodes, *B. xylophilus* and *M. incognita*. The four isolated constituents also displayed phototoxic activity against the nematodes. The results indicate that the ethanol extract of *N.* *incisum* and its four isolated constituents have potential for development into natural nematicides for control of plant-parasitic nematodes.

## 1. Introduction

Plant-parasitic nematodes are a serious worldwide threat to forestry and agriculture because of their wide range of host plants and short biological cycles. It has been estimated that plant parasitic nematodes have caused as much as $100 billion in annual losses of crops and plants worldwide [1]. The southern root-knot nematode (*Meloidogyne incognita* (Kofoid & White) Chitwood) is the most economically important and widely distributed nematode throughout China, and considerable crop loss is caused by this nematode [1]. The pine wood nematode (*Bursaphelenchus xylophilus* (Steiner & Buhrer) Nickle) causes pine wilt disease by inducing rapid wilting and leads to death of host pines [2]. Nematode management is generally based upon chemical treatments (soil fumigation, e.g., methylbromide and dichloropropane), but environmental concerns and governmental regulations are now resulting in a strong interest in nematicides of natural origin [3,4]. Plants are a prominent source of new nematicidal chemicals, since many plants have been reported to possess nematicidal activities [5,6,7,8,9,10,11,12,13,14]. A series of nematicidal substances of plant origin such as triglycerides, sesquiterpenoids, alkaloids, steroids, diterpenoids, monoterpenoids, and flavonoids have been identified [3,4,8,9,10,15,16,17,18,19].

During the screening program for new agrochemicals from Chinese medicinal herbs and wild plants, the ethanol extract of the rhizomes of *Notopterygium incisum* Ting ex H. T. Chang (Family: Umbelliferae) (Figure S1) was found to possess nematicidal toxicity against the two species of nematodes, *B. xylophilus* and *M. incognita*. *Notopterygium incisum* is perennial herb native to China (Gansu, Qinghai, Shaanxi, Sichuan province, and Tibet Autonomous Region). It grows at a range of 1600 to 5000 m above sea level and lives among forest edges and scrubs that border the grasslands of these elevated slopes [20]. The rhizomes of *N. incisum* known as “Qianghuo” in Chinese, is a well-known traditional Chinese medicinal herbs. They are used in herbal preparations for a series of symptoms from the common cold, chills, fever, rheumatoid arthritis, and general limb or body aches and pains [14]. Previous phytochemical investigations revealed that the rhizomes of *N. incisum* contained phenolic compounds, alkaloids, polyacetylenes, coumarins, sesquiterpenoids, and glycosides [21,22,23,24,25,26,27,28,29,30,31,32,33,34]. Pharmacological studies have revealed that its extracts or constituents possess anti-inflammatory, antioxidative, analgesic, and anti-influenza activity as well as antiproliferative activity against several cancer cell lines [21,22]. However, a literature survey shows that there is no report on nematicidal activity of the ethanol extract of *N. incisum* rhizomes. Thus, the objective of this study was to investigate the nematicidal activity of the ethanol extract of *N. incisum* rhizomes against the two species of nematodes and isolation of active constituents from the ethanol extract.

## 2. Results and Discussion

### 2.1. Isolated Bioactive Compounds

**Figure 1:**
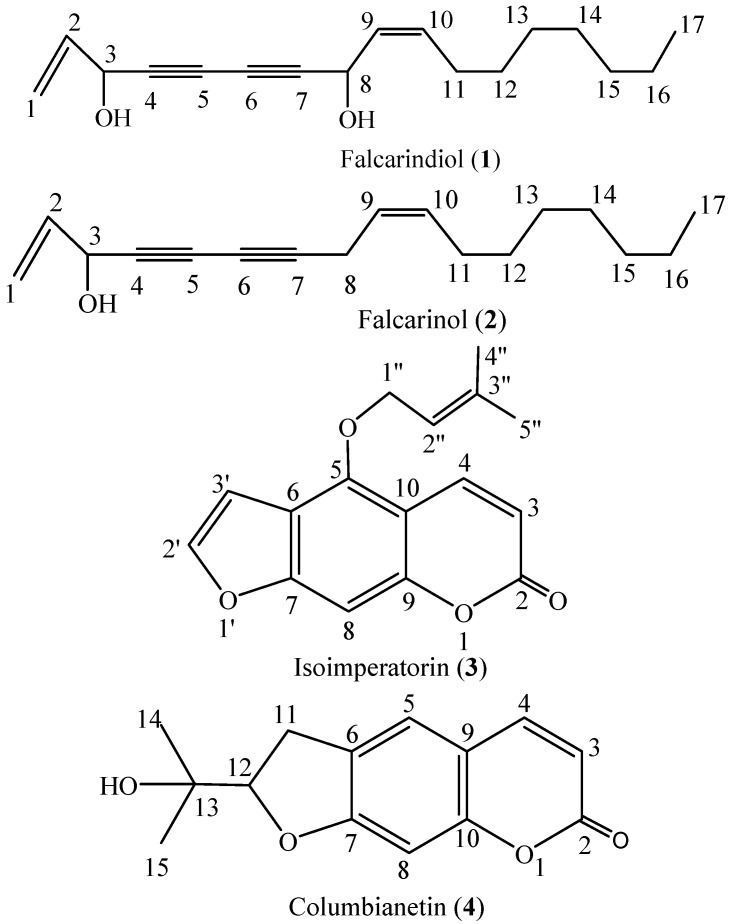
Constituent compounds isolated from the ethanol extract of *Notopterygium incisum* rhizomes.

Four bioactive compounds were isolated and based on bioassay-guided fractionation and identified based on their spectroscopic data and comparison with literature vales. Their chemical structures are given in Figure 1.

### 2.2. Nematicidal Activity

Among the four isolated constituents, falcarindiol and falcarinol exhibited strong nematicidal activity against the pine wood nematode (*B. xylophilus*) in dark with LC_50_ values of 2.20 µg/mL and 12.62 µg/mL, respectively. Isoimperatorin and columbianetin also had LC_50_ values of 21.83 µg/mL and 103.44 µg/mL against *B. xylophilus*, respectively (Table 1). Moreover, based on LC_50_ values, the two polyacetylene compounds demonstrated stronger nematicidal activity (no overlap of the 95% FLs) than a positive control, 2% avermectin (dark, LC_50_ = 3.57 µg/mL) and two coumarins (isoimperatorin and columbianetin) against the pine wood nematodes (Table 1). When using 12 h light and 15 min UV light treatment, the nematicidal activity of the four isolated constituents against *B. xylophilus* increased. For example, falcarindiol had LC_50_ values of 0.95 µg/mL (light) and 0.73 µg/mL (UV) against *B. xylophilus*, two-to-three times more toxic than in dark (Table 1) while falcarinol showed almost nine times more toxic in UV light treatment than in dark against *B. xylophilus.* Moreover, after 15 min UV light treatment, the two coumarins also possessed two-to-three times more toxic to the pine wood nematodes than the dark treatment.

Based on LC_50_ values, the two polyacetylene compounds (falcarindiol and falcarinol, in dark LC_50_ = 1.08 µg/mL and 4.96 µg/mL, respectively) exhibited stronger nematicidal activity (no overlap of the 95% FLs) than two coumarins (isoimperatorin and columbianetin, dark treatment LC_50_ = 7.57 µg/mL and 30.91 µg/mL, respectively) against the southern root-knot nematodes (*M. incognita*) (Table 2). Falcarindiol also possessed the same level of nematicidal activity against the southern root-knot nematodes as the positive control, 2% avermectin (in dark, LC_50_ = 1.25 µg/mL) and the three other constituents showed less toxic than the positive control (Table 2). When using 15 min UV light treatment, falcarindiol, falcarinol, and isoimperatorin demonstrated almost five times more toxic to the southern root-knot nematodes than in dark treatment while columbianetin showed only two times more toxic (Table 2).

In the previous reports, several naturally occurring polyacetylenes have been demonstrated to possess toxic or phototoxic activity against insects, especially larval mosquitoes [35,36,37,38,39,40]. Falcarinol and falcarindiol were also demonstrated to possess larvicidal activity against the common house mosquito (*Culex pipiens*) with 24 h LC_50_ values of 3.49 ppm and 6.51 ppm, respectively [41]. There were several reports on nematicidal activity of natural occurring polyacetylenes (derived from *Carthamus* *tinctorius*) against plant parasitic nematodes such as, rice white tip nematode (*Aphelencoides besseyi*) with more than 80% mortality after five days at a concentration of 10 ppm treatment [42,43]. Moreover, 14 polyacetylenes or their thiophene derivates, isolated from species of the Asteraceae were found to possess toxicity against adult nematodes (*Caenorhabditis elegans*) and toxicity of the compounds was found to be enhanced greatly by irradiation with near-UV radiation or natural sunlight [44]. However, their investigation with falcarindiol showed no photoactivation with UVA radiation [41]. Moreover, falcarinol and falcarindiol have been identified as antifungal compounds in many Apiaceae plant species inhibiting spore germination of different fungi in concentrations ranging from 20 to 200 μg/mL [45]. Falcarinol and falcarindiol also exhibited significant anti-*Candida*, antibacterial, and antimycobacterial activity, with an ability to kill *Mycobacterium tuberculosis* and isoniazid-resistant *Mycobacterium avium* at 10 μg/disk in a disk diffusion assay [46]. However, this is the first report of nematicidal activity and photoactivation with UV treatment of falcarinol and falcarindiol against the two species of nematodes. The mode of action of falcarinol and falcarindiol on nematodes was not investigated; however, the mechanism for antifungal activity of falcarinol and falcarindiol is believed to involve disruption of cell membranes [47]. Thus, falcarinol, the more lipophilic compound, was more toxic than falcarindiol against the two species of nematodes, the more polar acetylene (Table 1). However, it was also suggested that the potent insecticidal action of falcarinol might be related to the GABAergic block associated with the higher intake expected in herbivorous insects because GABA_A_ receptors are important targets of neuroactive pesticides [48,49].

Columbianetin and isoimperatorin had been demonstrated to possess insecticidal activity against several insects, such as the cabbage aphid (*Brevicoryne brassicae*), Egyptian cottonworm (*Spodoptera littoralis*), larvae of *Aedes albopictus*, *A. aegypti*, *Culex pipiens pallens* [50,51,52]. Columbianetin also exhibited antibacterial, antifungal, and cytotoxic activity [53,54,55]. However, this is first report of nematicidal activity against the two species of plant-parasitic nematodes.

The above findings suggest that nematicidal activity of the ethanol extract of *N. incisum* rhizomes and its four isolated constituents—especially the two polyacetylene compounds—against the two species of plant-parasitic nematodes is quite promising. As they are currently commonly used, nematicides are synthetic pesticides and these synthetic pesticides are also highly toxic to humans and other non-target organisms, the ethanol extract and its four isolated constituents show potential to be developed as possible natural nematicides for the control of *B. xylophilus* and *M. incognita*.

In traditional Chinese medicine, *N. incisum* rhizomes are used in herbal preparations for a series of symptoms from the common cold, chills, fever, rheumatoid arthritis, and general limb or body aches and pains [20]. It thus seems that this medicinal herb is quite safe for human consumption because it has been used as a medicinal herb for hundreds of years. Moreover, the four isolated constituents are naturally present in many vegetables and fruits, e.g., carrot, celery, parsley, coriander, ginseng, etc. Thus it seems that the four isolated constituents are quite safe for human consumption. However, no experimental data about the safety of this herb is available so far, so to develop a practical application for the ethanol extract and the isolated constituents as novel nematicides, further research on the safety of the extract/compounds to humans is needed.

## 3. Experimental

### 3.1. General

^1^H and ^13^C-NMR spectra were recorded on Bruker Avance ACF300 (300 MHz (1H)) and Bruker Avance AMX500 (500 MHz (1H), Bruker BioSpin AG, Fällanden, Switzerland) instruments using CDCl_3_ as the solvent with TMS as internal standard.

### 3.2. Plant Material and Extraction

The dried rhizomes of *N. incisum* (5 kg) were purchased from Anguo Chinese Medicinal Herbs Market (Anguo, Hebei, China), which harvested from Gansu Province, China. The species was identified by Dr QR Liu, College of Life Sciences, Beijing Normal University, Beijing, China. A voucher specimen of *N. incisum* (No. CMH-Qianghuo-Gansu-2015-08) was deposited at the museum of the Department of Entomology, China Agricultural University, Beijing, China.

The dried rhizomes were cut into pieces and then successively extracted with 40 L different concentrations of ethanol/ tap water mixtures (95%, 75%, 50%, by volume) and tap water at room temperature for three days. The extracts were then filtered, mixed and concentrated under vacuum to afford crude extract (800 g). After that, the crude extract was diluted with distilled water (2 L) and then successively partitioned with the same volume of *n*-hexane, chloroform, ethyl acetate and *n*-butyl alcohol. Each partition was concentrated separately to obtain four solvent fractions (27 g, 86 g, 31 g, 32 g) and stored in brown glass bottles at 4 °C for further experiments.

### 3.3. Bioassay-Directed Fractionation

The crude ethanol extract and its four solvent fractions were screened for their nematicidal potential against juveniles of the two species of nematodes as described below. On the basis of 72 h mortalities at a concentration of 1000 μg/mL, the most active (chloroform fraction) was subjected to further fractionation.

The chloroform fraction of *N. incisum* extracts was chromatographed on a silica gel (Merck grade 9385, 230–400 mesh, Darmstadt, Germany, 1000 g) column (85 mm i.d., 850 mm length), eluting with a solvent system (petroleum ether/ethyl acetate/ethanol, by volume) of increasing polarity. Column fractions were analyzed by TLC (precoated GF254 plate; Qingdao Marine Chemical Plant, Qingdao, China) profiles and similar fractions were combined to yield 12 fractions (Fa1–Fa14). Nematicidal activity bioassays of these fractions were evaluated at a concentration of 1000 μg/mL to afford four bioactive principles (Fa2, Fa4, Fa6, Fa7). Fa2 was submitted to preparative silica gel column chromatography (PTLC) (precoated GF254 plate) using petroleum ether/ethyl acetate (100:5, by volume) as an eluent to give isoimperatorin (15 mg). The other three fractions were further purified by silica gel (Merck grade 9385, 230–400 mesh, 300 g) column (10 mm i.d., 500 mm length) chromatography using a petroleum ether/ethyl acetate solvent system and by PTLC as well as by Sephadex LH-20 column, to get compounds with the following order: columbianetin (18 mg), falcarinol (25 mg), and falcarindiol (22 mg). The structure of the compounds was elucidated based on nuclear magnetic resonance (Figures S2–S5).

### 3.4. Nematodes

Eggs of *M. incognita* were extracted from infected roots of tomato (*Solanum lycopersicum* L.). All the tomatoes were reared in a growth chamber (16:8 h L:D, 25–28 °C, 75%–80% RH). When reaching the five-leaf stage, tomato plants were used for inoculations; and 43 days later, infected tomato plants were uprooted and the roots were washed free of soil with tap water. Egg masses were hand-picked using sterilized tweezers from infected roots and rinsed three times with sterilized distilled water. To obtain second-stage juveniles (J2) of *M. incognita*, egg masses were placed on a mesh nylon filter (openings 30 μm in diameter) [56]. J2s that passed through the filter were collected daily and used for bioassays immediately.

*B. xylophilus* was collected from sawdusts of infected pine woods in Fuling District, Chongqing City, China (29.70° N and 107.39° E) in September 2015, and obtained through the modified Baermann funnel technique [57]. Colonies of *B. xylophilus* were maintained on *Botrytis cinerea* cultures. The fungus *B. cinerea* was cultured on potato dextrose agar (PDA) in a growth chamber (25–28 °C in dark). When *B. cinerea* was fully grown on PDA, the plates were inoculated with *B. xylophilus* and cultured in the growth chamber (25–28 °C in dark) until the fungal mycelium were completely consumed by *B. xylophilus.* Then, *B. xylophilus* were collected using the modified Baermann funnel technique [48], washed with a mixture of 0.1% streptomycin sulfate and 0.002% actinone three times to remove any surface bacterial or fungal contaminants and immediately used for bioassays.

### 3.5. Nematicidal Activity Bioassays

Nematicidal activity bioassays of *M. incognita* were taken under laboratory conditions at 25–28 °C. The standard nematode suspensions of *M. incognita* were prepared by appropriate dilution with sterilized distilled water to get approximately 100 J2s/mL. All the tested extracts or compounds were dissolved in ethanol and diluted with distilled water to obtain stock solutions of double the treatment concentrations, which were determined by a series of range-finding tests. However, the final concentration of ethanol in each treatment never exceeded 1% (by volume). Then, each well of 24-well tissue culture plates were added with 500 μL standard J2 suspension. Numbers of active J2 in every well were counted under a stereoscope at 10× and 5× before 500 μL stock solution was added to the corresponding well. Plates were then covered with rice paper to avoid evaporation. Each test was composed of five concentrations with three replicates. Commercial avermectin (purchased from Aladdin-Reagent Company, Shanghai, China)—serving as a positive control and distilled water containing ethanol (1%, by volume)—was used as a negative control. Both treated and control J2 nematodes were placed in a growth chamber at 25–28 °C in dark or in light. To measure photoactive effect, 15 min UV radiation was provided and the treated sets were placed to the growth chamber at 25–28 °C in dark. Mortality recordings were taken 72 h after treatment. J2 nematodes that showed no movements when stimulated with a fine needle were considered to be dead.

Nematicidal activity bioassays of *B. xylophilus* were taken as almost the same as *M. incognita* bioassays, but only juveniles were used. 

### 3.6. Isolated Constituent Compounds

*Falcarindiol* (**1**, Figure 1). Brownish oil, ^1^H-NMR (CDCl_3_, 500 MHz) δ (ppm): δ 6.00–5.92 (1H, m, H-2), 5.64 (1H, dd, *J* = 17.8, 7.7 Hz, H-10), 5.52 (2H, dd, *J* = 24.3, 13.4 Hz, H-1), 5.23 (1H, d, *J* = 8.3 Hz, H-8), 4.97 (1H, d, *J* = 4.8 Hz, H-3), 2.13 (2H, q, *J* = 7.4 Hz, H-11), 1.44–1.37 (3H, m, H-12), 1.29 (8H, d, *J* = 10.5 Hz, H-13, 14, 15, 16), 0.90 (3H, t, *J* = 6.4 Hz, H-17). ^13^C-NMR (CDCl_3_, 125 MHz) δ (ppm): 135.8 (C-2), 134.7 (C-10), 127.6 (C-9), 117.4 (C-1), 78.8 (C-7), 78.4 (C-4), 72.4 (C-5), 63.6 (C-6), 63.5 (C-3), 58.6 (C-8), 31.8 (C-11), 29.28–29.07 (C-12, 13, 14), 27.8 (C-15), 22.7 (C-16), 14.1 (C-17). The data matched with previous reports [58,59].

*Falcarinol* (**2**, Figure 1). Yellow oil, ^1^H-NMR (CDCl_3_, 500 MHz) δ (ppm): 5.96 (1H, ddd, *J* = 17.0, 10.1, 5.4 Hz, H-10), 5.53–5.50 (1H, m, H-10), 5.47 (1H,s, H-1), 5.40 (1H, d, *J* = 10.5 Hz, H-9), 5.28–5.24 (1H, m, H-1), 4.94 (1H, d, *J* = 5.3 Hz, H-3), 3.06 (2H, dd, *J* = 6.9, 0.7 Hz, H-8), 2.06 (1H, d, *J* = 7.8 Hz, H-3), 2.04 (1H, s, H-11), 1.40–1.36 (2H, m, H-12), 1.31–1.29 (8H, m, H-13, 14, 15, 16), 0.90 (3H, t, *J* = 6.9 Hz, H-17). ^13^C-NMR (CDCl_3_, 125 MHz) δ (ppm): 136.1 (C-2), 133.1 (C-10), 121.9 (C-9), 117.1 (C-1), 80.3 (C-7), 74.2 (C-4), 71.3 (C-5), 64.0 (C-6), 63.6 (C-3), 31.8 (C-15), 29.2 (C-12, 13, 14), 27.2 (C-11), 22.6 (C-16), 17.7 (C-8), 14.1 (C-17). The data matched with previous reports [58,59].

*Isoimperatorin* (**3**, Figure 1). Crystal, ^1^H-NMR (CDCl_3_, 500 MHz) δ (ppm): 8.18 (1H, d, *J* = 9.8 Hz, H-4), 7.62 (1H, s, H-2′), 7.18 (1H, s, H-8), 6.98 (1H, s, H-3′), 6.29 (1H, d, *J* = 9.7 Hz, H-3), 5.56 (1H, t, *J* = 6.4 Hz, H-2″), 4.94 (2H, d, *J* = 7.0 Hz, H-1′), 1.82 (3H, s, H-4″), 1.72 (3H, s, H-5″). ^13^C-NMR (CDCl_3_, 125 MHz) δ (ppm): 161.4 (C-2), 158.2 (C-7), 152.7 (C-9), 149.0 (C-5), 144.9 (C-2′), 139.8 (C-3′′), 139.7 (C-4), 119.1 (C-2″), 114.3 (C-6), 112.5 (C-3), 107.6(C-10), 105.0 (C-3′), 94.3 (C-8), 69.8 (C-1″), 25.8 (C-4″), 18.2 (C-5″). The data matched with previous reports [50].

*Columbianetin* (**4**, Figure 1). Crystal, ^1^H-NMR (MeOD, 500 MHz) δ (ppm): δ 7.88 (1H, d, *J* = 9.5 Hz, H-4), 7.43 (1H, s, H-5), 6.75 (1H, s, H-8), 6.22 (1H, d, *J* = 9.5 Hz, H-3), 4.78 (1H, t, *J* = 8.7 Hz, H-12), 3.28 (2H, d, *J* = 9.6 Hz, H-11), 1.32 (3H, s, H-14), 1.26 (3H, s, H-15). ^13^C-NMR (MeOD, 125 MHz) δ (ppm): 163.9 (C-7), 162.4 (C-2), 155.5 (C-10), 144.9 (C-4), 125.9 (C-6), 123.6 (C-5), 112.7 (C-9), 110.8 (C-3), 96.8 (C-8), 91.1 (C-12), 70.9 (C-13), 28.9 (C-11), 24.0 (C-14), 23.9 (C-15). The data matched with previous reports [59,60].

### 3.7. Data Analysis

Data were corrected for control mortality using Abbott’s formula [61]. LC_50_ and LC_90_ values, along with 95% confidence limits (CLs), and *chi*-square values were calculated using SPSS 14.0 software. LC_50_ values of the tested materials were considered to be significantly different when their 95% confidence limits (CLs) failed to overlap. *Chi*-square values were significant at the *p* < 0.05 level.

## 4. Conclusions

The present work indicated that the ethanol extract of *N. incisum* rhizomes and its four isolated constituents demonstrated strong nematicidal activity against *B. xylophilus* and *Meloidogyne incognita.* Our results suggested that the extract of *N. incisum* rhizomes and its four isolated constituents, especially falcarinol and falcarindiol, have potential for development into natural nematicides for control of plant-parasitic nematodes.

## Figures and Tables

**Table 1:** Nematicidal activity of bioactive compounds from *Notopterygium incisum* rhizomes against *Bursaphelenchus xylophilus*.

Compound	Treatment	LC_50_ (μg/mL) (95% FL) *	RT **	LC_90_ (μg/mL) (95% FL) *	Slope ± SE
Columbianetin	Dark	103.44 (84.52–123.10)	-	375.11 (288.16–560.48)	2.29 ± 0.21
	Light	72.12 (59.98–83.82)	1.43	268.50(217.65–361.20)	2.25 ± 0.24
	UV	32.11 (25.05–38.74)	3.22	85.64 (67.29–128.53)	3.01 ± 0.34
Falcarindiol	Dark	2.20 (1.82–2.61)	-	11.82 (8.66–18.82)	1.76 ± 0.20
	Light	0.95 (0.54–1.30)	2.32	13.97 (7.82–48.18)	1.10 ± 0.21
	UV	0.73 (0.62–0.83)	3.01	2.65 (2.17–3.50)	2.29 ± 0.32
Falcarinol	Dark	12.61 (9.82–15.19)	-	69.87 (54.61–100.05)	1.72 ± 0.20
	Light	7.42 (4.93–9.66)	1.69	30.79 (21.45–63.14)	1.63 ± 0.30
	UV	1.43 (1.15–1.71)	8.82	3.98 (3.18–5.54)	2.88 ± 0.23
Isoimperatorin	Dark	21.83 (16.66–27.54)	-	236.64 (135.22–660.45)	1.24 ± 0.20
	Light	15.14 (9.05–20.59)	1.44	250.35 (128.36–1071.92)	2.25 ± 0.21
	UV	12.07 (10.13–13.86)	1.81	35.90 (30.39–45.16)	2.71 ± 0.29
Ethanol extract	Dark	45.21 (40.12–49.23)	-	234.67 (211.56–256.78)	2.23 ± 0.24
Avermectin		0.07 (0.06–0.08)	-	0.24 (0.21–0.26)	2.45 ± 0.20

* Fiducial limits; ** Relative toxicity = LC_50_ value in dark treatment/LC_50_ values (in light or UV).

**Table 2:** Nematicidal activity of bioactive compounds from *Notopterygium incisum* rhizomes against *Meloidogyne incognita*.

Compound	Treatment	LC_50_ (μg/mL) (95% FL) *	RT **	LC_90_ (μg/mL) (95% FL) *	Slope ± SE
Columbianetin	Dark	30.91 (26.04–36.18)	-	161.08 (124.53–227.93)	1.79 ± 0.16
	Light	28.29 (14.78–40.44)	1.09	171.12 (138.24–262.45)	1.61 ± 0.27
	UV	12.33 (9.45–14.85)	2.51	43.09 (35.06–58.65)	2.36 ± 0.31
Falcarindiol	Dark	1.08 (0.89–1.28)	-	5.99 (4.36–9.68)	1.72 ± 0.20
	Light	0.56 (0.44–0.67)	1.93	2.33 (1.79–3.47)	2.07 ± 0.19
	UV	0.22 (0.15–0.29)	4.91	2.31 (1.54–4.65)	1.26 ± 0.09
Falcarinol	Dark	4.96 (4.20–5.76)	-	24.00 (19.10–32.27)	1.87 ± 0.15
	Light	3.44 (2.95–3.90)	1.44	10.55 (9.11–12.74)	2.63 ± 0.23
	UV	1.00 (0.86–1.15)	4.96	5.50 (4.12–8.30)	1.73 ± 0.17
Isoimperatorin	Dark	17.21 (14.86–19.85)	-	74.55 (58.88–101.81)	1.79 ± 0.19
	Light	7.57 (4.52–10.30)	2.27	125.18 (64.18–535.96)	1.05 ± 0.13
	UV	3.30 (1.96–4.46)	5.22	18.22 (14.41–26.25)	1.73 ± 0.17
Ethanol extract	Dark	22.34 (19.89–24.67)	-	130.56 (119.25–142.79)	1.56 ± 0.14
2% Avermectin	–	0.03 (0.02–0.03)	-	0.24 (0.22–0.27)	2.08 ± 0.16

* Fiducial limits; ** Relative toxicity = LC_50_ value in dark treatment/LC_50_ values (in light or UV).

## Supplementary Materials

Supplementary materials can be accessed at: http://www.mdpi.com/1420-3049/21/10/1276/s1.
