# Supplementary material for: Identification of Nematicidal Constituents of Notopterygium incisum Rhizomes against Bursaphelenchus xylophilus and Meloidogyne incognita
Source: Molecules. 2016 Sep 23;21(10):1276. doi: 10.3390/molecules21101276 (PMC6274203; doi:10.3390/molecules21101276)
Supplement: Supplementary file 1 [file molecules-21-01276-s001.pdf]

Supplementary Materials: Identification of Nematicidal Constituents of *Notopterygium incisum* Rhizomes against *Bursaphelenchus xylophilus* and *Meloidogyne incognita*

Gai Liu, Daowan Lai, Qi Zhi Liu, Ligang Zhou and Zhi Long Liu

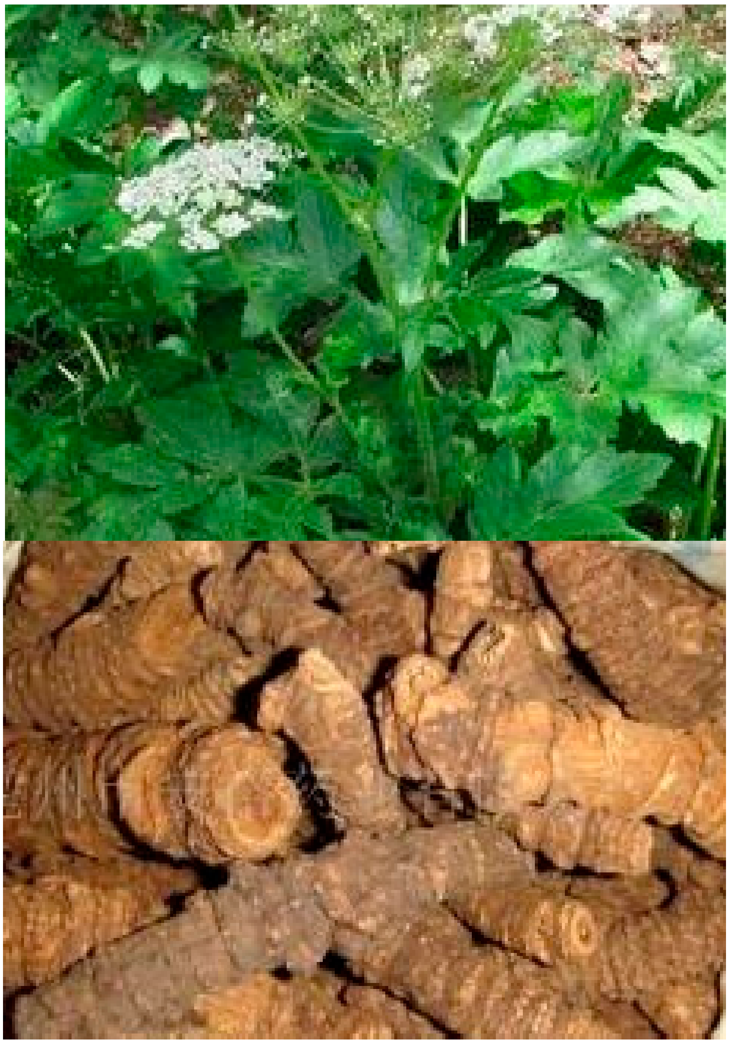

Figure S1. Picture of *Notopterygium incisum*.

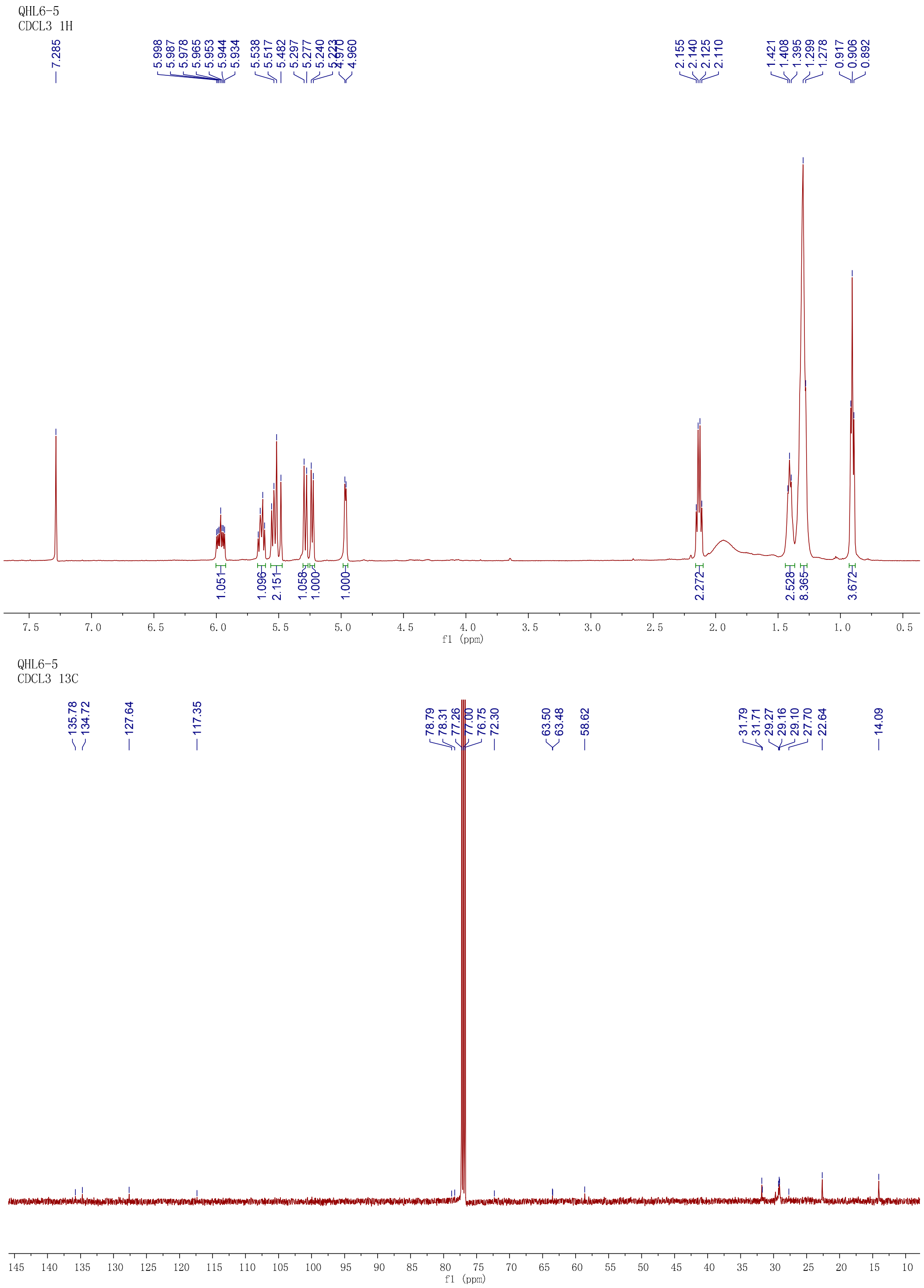

Figure S2. ^{1}H-NMR, ^{13}C-NMR spectra of falcarindiol.

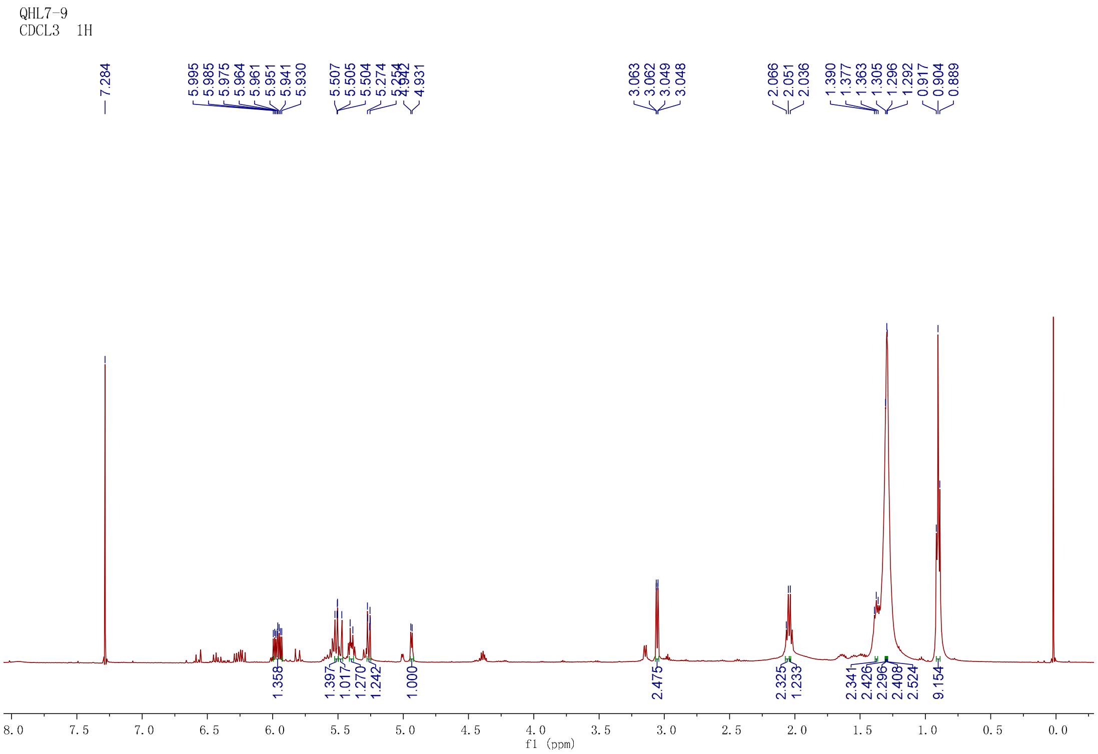

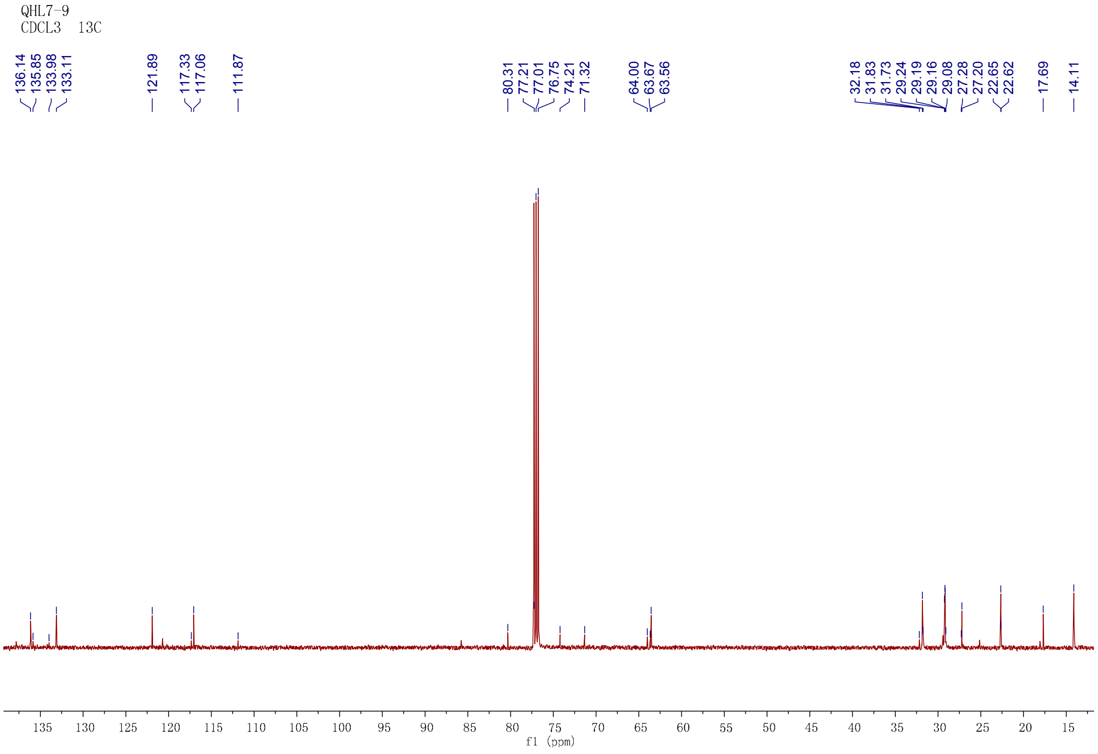

Figure S3. ^{1}H-NMR, ^{13}C-NMR spectra of falcarinol.

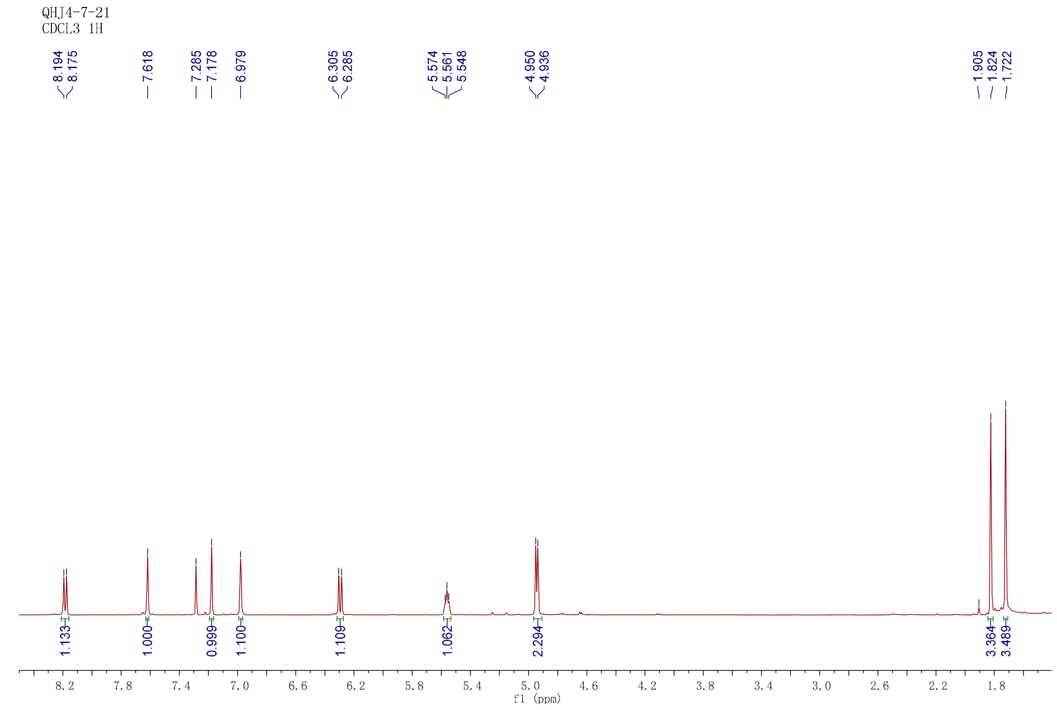

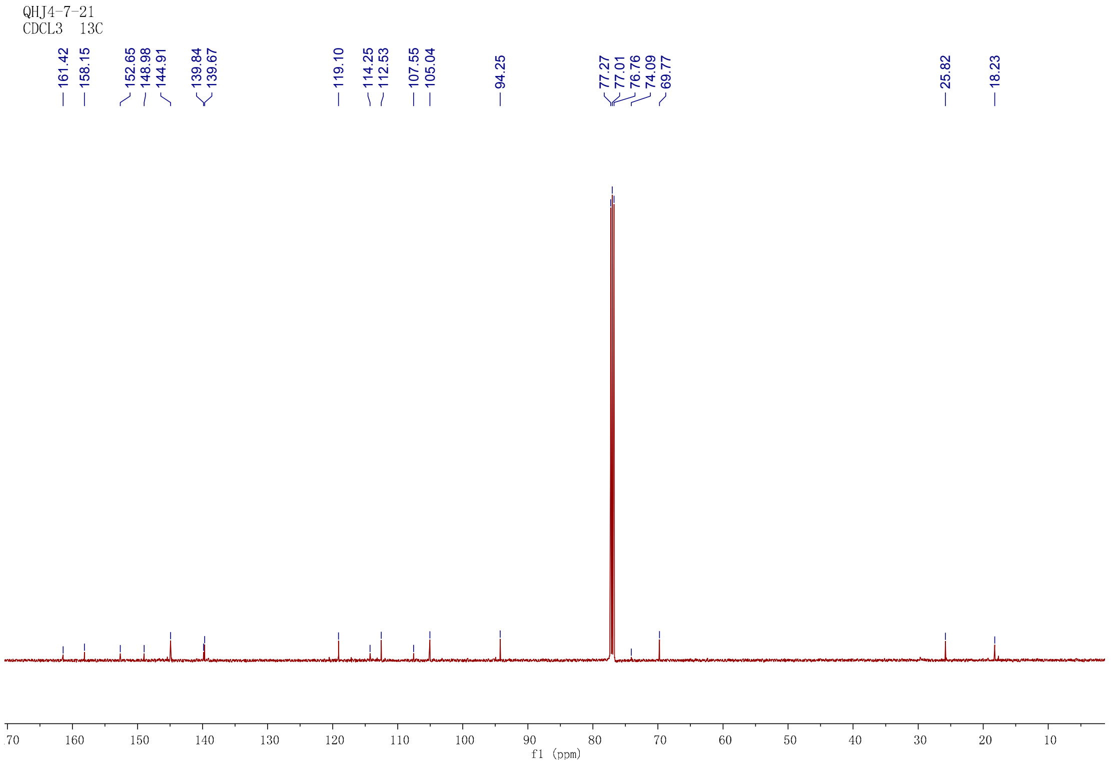

Figure S4. ^{1}H-NMR, ^{13}C-NMR spectra of isoimperatorin.

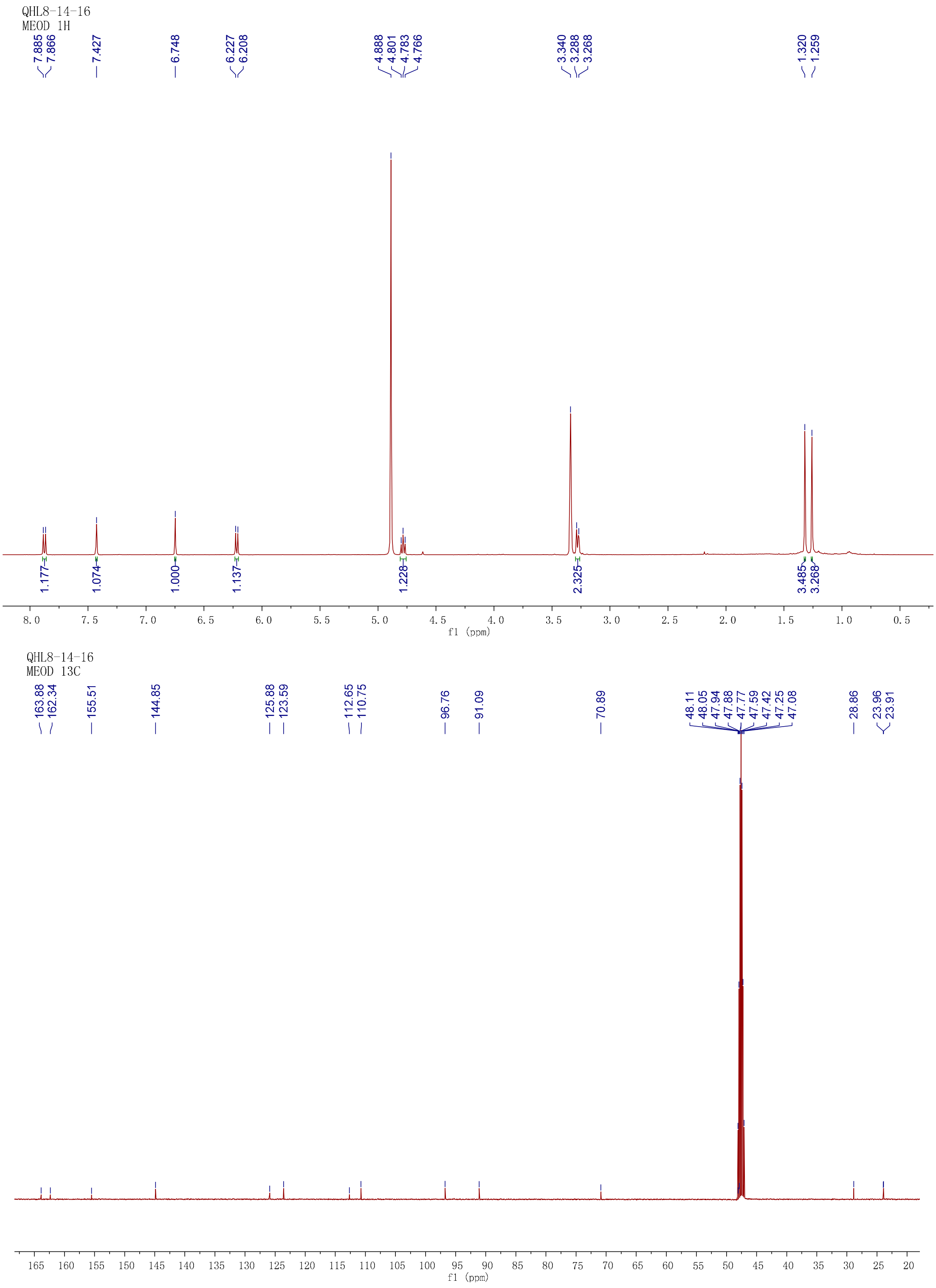

Figure S5. ^{1}H-NMR, ^{13}C-NMR spectra of columbianetin.
